# *Wolbachia’s* Deleterious Impact on *Aedes aegypti* Egg Development: The Potential Role of Nutritional Parasitism

**DOI:** 10.3390/insects11110735

**Published:** 2020-10-27

**Authors:** Megan J. Allman, Johanna E. Fraser, Scott A. Ritchie, D. Albert Joubert, Cameron P. Simmons, Heather A. Flores

**Affiliations:** 1 Institute of Vector-Borne Disease, Monash University, Melbourne 3800, Australia; Johanna.Fraser@monash.edu (J.E.F.); cameron.simmons@worldmosquito.org (C.P.S.); Heather.Flores@monash.edu (H.A.F.); 2 Department of Microbiology, Monash University, Melbourne 3800, Australia; 3 World Mosquito Program, Monash University, Melbourne 3800, Australia; scott.ritchie@worldmosquito.org (S.A.R.); albert.joubert@worldmosquito.org (D.A.J.); 4 College of Public Health, Medical and Veterinary Sciences, James Cook University, Smithfield, QLD 4811, Australia; 5 Australian Institute of Tropical Health and Medicine, James Cook University, Smithfield, QLD 4811, Australia; 6 Oxford University Clinical Research Unit, Hospital for Tropical Diseases, Ho Chi Minh City 710400, Vietnam

**Keywords:** *Wolbachia*, *Aedes aegypti*, biocontrol, nutritional parasitism

## Abstract

**Simple Summary:**

Mosquito-borne viral diseases such as dengue, Zika and chikungunya cause a significant global health burden and are currently increasing in outbreak frequency and geographical reach. *Wolbachia pipientis*, an endosymbiotic bacterium, offers a solution to this. When *Wolbachia* is introduced into the main mosquito vector of these viruses, *Aedes aegypti*, it alters the mosquito’s reproductive biology, as well as reducing the ability of the mosquitoes to transmit viruses. These traits can be leveraged to reduce virus transmission within a community by mass releasing *Wolbachia*-infected mosquitoes. However, *Wolbachia* has some negative effects on *Aedes aegypti* fitness, particularly egg longevity, and the reason behind this remains ambiguous. Insect fitness is very important for the success for *Wolbachia*-biocontrol strategies as they rely on the released insects being competitive with the wild mosquito population. This review summarises the fitness effects of *Wolbachia* on *Aedes aegypti* and investigates the possible contribution of *Wolbachia* as a nutritional parasite in lowering host fitness. It proposes the next stages of research that can be conducted to address nutritional parasitism to aid in the expansion of *Wolbachia*-based disease management programs worldwide.

**Abstract:**

The artificial introduction of the endosymbiotic bacterium, *Wolbachia pipientis,* into *Aedes (Ae.) aegypti* mosquitoes reduces the ability of mosquitoes to transmit human pathogenic viruses and is now being developed as a biocontrol tool. Successful introgression of *Wolbachia*-carrying *Ae. aegypti* into native mosquito populations at field sites in Australia, Indonesia and Malaysia has been associated with reduced disease prevalence in the treated community. In separate field programs, *Wolbachia* is also being used as a mosquito population suppression tool, where the release of male only *Wolbachia*-infected *Ae. aegypti* prevents the native mosquito population from producing viable eggs, subsequently suppressing the wild population. While these technologies show great promise, they require mass rearing of mosquitoes for implementation on a scale that has not previously been done. In addition, *Wolbachia* induces some negative fitness effects on *Ae. aegypti*. While these fitness effects differ depending on the *Wolbachia* strain present, one of the most consistent and significant impacts is the shortened longevity and viability of eggs. This review examines the body of evidence behind *Wolbachia’s* negative effect on eggs, assesses nutritional parasitism as a key cause and considers how these impacts could be overcome to achieve efficient large-scale rearing of these mosquitoes.

## 1. Mosquito Biocontrol Methods

Mosquito-borne diseases are a growing global health and economic burden, with one-third of the world’s population currently at risk of dengue infection [1,2]. Incidence of dengue fever has increased 30-fold within the last 50 years and is endemic in more than 100 countries, with an estimated 390 million infections occurring annually, 20,000 of which are fatal [3,4,5]. The *Aedes (Ae.) aegypti* mosquito is the primary vector of dengue virus (DENV) and other prevalent human pathogens including Zika virus (ZIKV) and chikungunya virus (CHIKV) [6,7]. Growing urban environments are enabling the expansion of these anthropophilic mosquitoes and rising global temperatures will likely increase the environmental suitability of mosquito vectors over time, increasing human exposure to disease vectors [8]. As such, the need for effective disease prevention methods is growing.

The release of *Wolbachia*-infected mosquitoes offers a promising vector management strategy. This technology manipulates vector activity by releasing mosquitoes infected with *w*Mel or *w*AlbB strain to either suppress or introgress into target insect populations. *Wolbachia* is an endosymbiotic bacterium that is maternally transmitted and estimated to exist in approximately 40–66% of insect species [9,10,11,12]. *Wolbachia* is not naturally present in *Ae. aegypti,* so it must be transinfected from another insect species [13,14]. Novel *Wolbachia* infections can induce changes in vector competency and host fitness [15]. *Wolbachia*-induced physiological manipulations, such as cytoplasmic incompatibility (CI) and pathogen protection, are harnessed in biological control methods. CI occurs when *Wolbachia*-infected males mate with uninfected females, resulting in embryonic lethality of offspring [16,17,18]. *Wolbachia*-infected females can rescue the lethal phenotype to successfully reproduce with both infected and uninfected males and produce *Wolbachia*-infected offspring, giving them a reproductive advantage over uninfected females. Population suppression using *Wolbachia*, known as the incompatible insect technique (IIT), involves the release of male mosquitoes that carry *Wolbachia* and leverages CI to reduce the number of viable mosquito offspring within a population. Alternatively, population introgression involves the release of both male and female *Wolbachia*-infected *Ae. aegypti* to mate with the native mosquito population where, due to CI, males prevent the further breeding of uninfected mosquitoes, while females pass *Wolbachia* to offspring due to maternal transmission [17,18]. *Ae. aegypti* mosquitoes that carry *Wolbachia* hold lower transmission potential for viruses such as DENV [19,20,21], ZIKV [22,23] and CHIKV [19,22,24], reducing the disease burden within communities once wild type mosquito populations have been replaced [25,26]. Both *Wolbachia*-based methods are dependent on the mass release of mosquitoes [26,27,28,29]. Success of these programs is, therefore, highly dependent on the large-scale production of quality release material to ensure that insects are competitive within the ecosystem which they are released into [30]. To ensure large-scale success of these programs, it is important to assess and understand the impact of *Wolbachia* on mosquito fitness. The fitness effects of *Wolbachia* have been well documented; however, the mechanism behind these effects remains ambiguous.

This review summarises the impact of different *Wolbachia* strains on *Ae. aegypti* egg development and longevity and examines the potential effects of *Wolbachia* acting as a nutritional parasite. The shelf-life of eggs is important for mass release programs because typically eggs are produced, stored and transported to sites prior to release or rearing for further production. This process can take time and subject eggs to variable environmental conditions, potentially compromising output quality. Understanding the impacts of *Wolbachia* on its host will help to inform the nutritional requirements of *Wolbachia*-infected insects and potentially improve the quality, shelf-life and field performance of *Wolbachia*-infected eggs produced for mass release programs.

## 2. Impacts of *Wolbachia* on Host Fitness

Eight different *Wolbachia* strains have been successfully transferred from various *Drosophila (D.)* or *Ae. albopictus* hosts into *Ae. aegypti* (*w*Mel, *w*MelPop-CLA, *w*MelCS, *w*Ri, *w*Au, *w*AlbA, *w*AlbB and *w*Pip) [13,15,31,32,33]. In *Wolbachia* introgression approaches, *w*Mel (native to *D. melanogaster*) and *w*AlbB (native to *Ae. albopictus*) are the current strains being utilised, with extensive literature testing their applications within the lab and field, while *w*MelPop-CLA (*Drosophila melanogaster Wolbachia* strain that was adapted to mosquitoes by passaging through an *Ae. aegypti*-derived cell line to create *w*MelPop-CLA [34]) was considered in early trials only [35]. In *Ae. aegypti,* all three strains induce complete CI and nearly perfect maternal transmission and effectively inhibit arboviruses [13,19,22,24,31,32,33,36,37,38]. While these strains have been found to successfully reduce vector competence and be self-sustaining under laboratory settings, negative fitness effects of *w*MelPop have impacted introgression success in native populations [39], and *w*AlbB and *w*Mel have been shown to reduce the longevity of eggs in their quiescent state. It is, therefore, important to understand the fitness effects of *Wolbachia* on its *Ae. aegypti* host and how this impacts application in the field.

### 2.1. Wolbachia Impacts on Host Fitness during Aquatic and Adult Life Stages

*Wolbachia* strains have varying levels of pathogenicity within *Ae. aegypti* (Table 1). The fitness effect of *w*Mel is minimal in both its natural host, *D. melanogaster*, and *Ae. aegypti* [33]. The introduction of *w*Mel into *Ae. aegypti* does not negatively impact adult female and male longevity compared to uninfected lines [15] and has minimal impact on mosquito fecundity. However, this does not prevent successful introgression of *w*Mel into wild populations after a stable equilibrium is reached [25,40,41,42,43,44]. A slight delay in larval developmental time has been observed when *w*Mel-infected and uninfected lines are reared within the same tray; however, overall hatching rates did not differ [45]. *w*AlbB causes minor negative effects on egg and adult longevity when introduced to *Ae. aegypti* [33,46,47] but has no impact on fecundity, larval development or mating success compared to wild type mosquitoes. In contrast, *w*MelPop-CLA is highly virulent in *Ae. aegypti*, decreasing adult female lifespan as well as blood-feeding success, fecundity, fertility and viability of eggs over time [13,32,48,49,50].

Field releases of *Ae. aegypti* carrying different *Wolbachia* strains for introgression have been conducted in multiple cities across the globe. *w*MelPop-CLA was released at sites in Australia and Vietnam. However, *Wolbachia* quickly dropped out of these populations after the completion of releases, most likely because these mosquitoes were substantially less fit than the native mosquito population [39]. *w*AlbB has been successfully established in mosquito populations in sites within Malaysia [26] and *w*Mel within sites in the Pacific Islands, Mexico, Colombia, Australia, Brazil and Indonesia [25,28,41,43,44]. These two strains have shown great promise to date, effectively introgressing into natural populations and reducing disease burden, but now a massive upscaling is required by these programs in order to have benefits city- and country-wide [26,28,43]. While the fitness effects on the aquatic and adult stage of these mosquitoes have been minimal for *w*Mel and *w*AlbB, the same cannot be said for egg survival.

### 2.2. Wolbachia Impacts on Egg Survival

Multiple studies have shown that *Wolbachia* strains *w*Mel, *w*AlbB and *w*MelPop-CLA reduce the lifespan of infected *Ae. aegypti* eggs compared to uninfected eggs, which can significantly impact the effectiveness of mosquito production strategies [15,47,52]. Mosquito eggs undergo quiescence (a form of dormancy) during periods of unfavourable environmental conditions, such as dry weather. The viability of eggs is maintained through a reduction of metabolic processes and resistance to desiccation [53,54]. When *w*Mel or *w*AlbB are present in an Australian or Brazilian *Ae. aegypti* background, the viability of *Wolbachia*-infected eggs decreases significantly faster than uninfected controls after a 30-day storage period [15,46,47,52]. Farnesi et al. [52] compared egg development and viability of Brazilian *w*Mel-*Ae. aegypti* with Brazilian wild type and a laboratory wild type strain and found that *w*Mel infection also delayed embryogenesis and decreased desiccation resistance through delayed eggshell formation. Morphological analysis identified a delay during germ band extension [52], which is when the serosal cuticle (responsible for desiccation resistance) is formed [55]. The virulent strain *w*MelPop-CLA similarly impacts *Ae. aegypti* egg viability, with studies indicating a 25–50% decline in hatch rate relative to uninfected controls over a 10–20-day period, depending on storage temperature [49,50]. It is evident that the presence of even the least virulent strains of *Wolbachia* impacts egg development and viability over time. Consequentially, a concern for mass production is that even minor fitness costs of *Wolbachia* could be amplified in a mass rearing setting. Improving these fitness effects—for example, by modifying artificial blood-feeding regimes and/or egg transport processes—could help to advance rearing and release strategies, generating major efficiency and cost benefits.

## 3. *Wolbachia* Genome Indicates Its Dependence on Host Resources

Multiple studies have shown that *Wolbachia’s* reduced genome, lacking amino acid and lipid biosynthesis genes, results in nutritional competition between *Wolbachia* and its host [56,57,58,59]. Wu et al. [56] sequenced the *w*Mel genome and compared it with closely related species of intracellular bacteria, such as Rickettsia, to understand the unique behaviour of *Wolbachia* within its host. This study identified a high proportion of amino acid uptake transporters alongside the presence of pathways for the catabolism of amino acids (including cysteine, glutamate, glutamine, proline, serine and threonine) in the *w*Mel genome, suggesting that amino acids are easily obtained from the host environment as a key source of energy for *w*Mel. Similarly, Foster et al. [57] sequenced the genomes of *w*Mel and *w*Bm (a *Wolbachia* strain native to *Brugia malayi,* a species of roundworm) and found evidence of nucleotide and select coenzyme biosynthesis pathways coinciding with a loss of amino acid biosynthesis pathways. In an attempt to develop an axenic *Wolbachia* culture, Krafsur et al. [60] found that amino acid concentrations in phenotype microarrays impact *Wolbachia*’s metabolic activity and can aid extracellular survival, indicating a high dependence on externally sourced amino acids for energy production. Together, these studies suggest that *w*Mel’s ability to produce amino acids is limited, yet it contains the necessary components to easily obtain amino acids from its host.

Genome studies have also identified gene losses that render *w*Mel unable to synthesise essential components of the bacterial cell wall. Wu et al. [56] identified a lack of biogenesis genes linked to the machinery that produces lipopolysaccharide components and alanine racemase, an enzyme that catalyses the conversion of L-alanine to D-alanine. Lipopolysaccharides function as a protectant and structural component in the outer membrane of Gram-negative bacteria such as *Wolbachia* [61], and the lipid component of lipopolysaccharide, lipid A, cannot be synthesised by *Wolbachia* [57]. D-alanine is also an important strengthening component of bacterial cell walls as it is a constituent of peptidoglycan, a strong and elastic polymer [62]. The structural necessity of lipopolysaccharide and D-alanine for proteobacterial membranes, combined with evidence that *w*Mel cannot produce these components itself, indicates that it is sequestering the necessary lipid and amino acid components from its insect host. These findings are further supported by Jiménez et al. [59], who compared the genomes of *w*Mel, *w*MelPop, *w*AlbB and *w*VitA (native to wasp species *Nasonia vitripennis*) and identified a lack of genes for the biosynthesis of antibiotic precursors, lipopolysaccharide biosynthesis (specifically lipid A) and amino acid metabolism (namely alanine, serine and glycine). This reduced genome of *Wolbachia* strongly indicates that the bacterium is unable to synthesise some essential nutrients and must therefore source them from its host.

## 4. Nutrient Quantification Studies

In support of these genome studies, the presence of *Wolbachia* in *Ae. aegypti* has been linked to changes in amino acid, lipid and carbohydrate compositions within adult mosquitoes. Nutrients such as these play a critical role in insect egg production, providing energy and physical structural elements and stimulating signalling pathways [63,64]. Comparative studies have been undertaken using nuclear magnetic resonance and lipid quantification techniques to measure the difference between amino acids and lipids in *Wolbachia*-infected (*w*Mel and *w*MelPop-CLA) and uninfected adult *Ae. aegypti* [65]. *Wolbachia* has been found to both increase (methionine, lysine, tryptophan) and decrease (histidine) levels of amino acids within its host. Similarly, lipid components such as acetate (a key constituent of fatty acids) and O-acetylcarnitine (synthesised from methionine and lysine) are increased while cholesterol and 3-hydroxyisovalerate are decreased [65]. Moreover, 3-hydroxyisovalerate is a fatty acid synthesised by bacterial species and requires lysine; hence, its decrease suggests that *Wolbachia* is potentially utilising lysine and restricting availability to other bacteria. Molloy et al. [66] analysed the lipid profile of *Ae. albopictus* cells and found that, in the presence of *Wolbachia*, there is a depletion of various lipid classes such as sphingolipids, diacylglycerols and phosphatidylcholines. These changes occurred alongside increased perturbations in vesicular trafficking, a key regulatory role of sphingolipids [67], which could alter lipid transportation and metabolism. Similarly, Koh et al. [68] conducted LC-MS on *w*Mel infected and uninfected *Ae. aegypti* to find a *Wolbachia*-induced decrease in six lipids: triacylglyceride, four phosphoethanolamines and a sphingolipid called glucosylceramide. Caragata et al. [69] also showed that *w*Mel and *w*MelPop-CLA-infected *Ae. aegypti* adults have 25% lower total cholesterol compared to uninfected control lines. Similarly, Geoghegan et al. [70] measured levels of esterified cholesterol (formed during storage and transport) and free cholesterol in *Ae. aegypti* cells infected and uninfected with *w*MelPop-CLA. Results indicated that esterified cholesterol levels were higher while free cholesterol levels were lower in *w*MelPop-infected cells. Therefore, this *Wolbachia* strain may upregulate cholesterol storage and transport but decrease overall available cholesterol in mosquito hosts. Further evidence of this is presented by Manokaran et al. [71], who used LC-MS lipidome analysis to show that acyl-carnitines are inhibited in the presence of *w*Mel in *Ae. aegypti*. Acyl-carnitines are a class of lipids that transport fatty acids into the mitochondria; therefore, *w*Mel-induced suppression potentially reduces lipid transport into the host organelles, increasing availability to *Wolbachia*. These studies suggest that *Wolbachia* is both influenced by host nutritional status and is influencing metabolism and sequestering a multitude of amino acids and lipids from its host, potentially impacting host nutritional requirements and having downstream negative effects on egg production and longevity.

The *Wolbachia*–host relationship is complicated and differs between bacterial strains and host species. While *Wolbachia* has also been observed to act as a nutritional mutualist in some cases [72], evidence for the strains introduced into *Ae. aegypti* indicates that they are highly dependent on host nutrients. Understanding the nutritional requirement for egg production could shed light on *Wolbachia*-induced fitness loss and effective mitigation methods.

## 5. Nutritional Requirements for Egg Production

### 5.1. Process of Egg Production in Aedes aegypti

**Figure 1:**
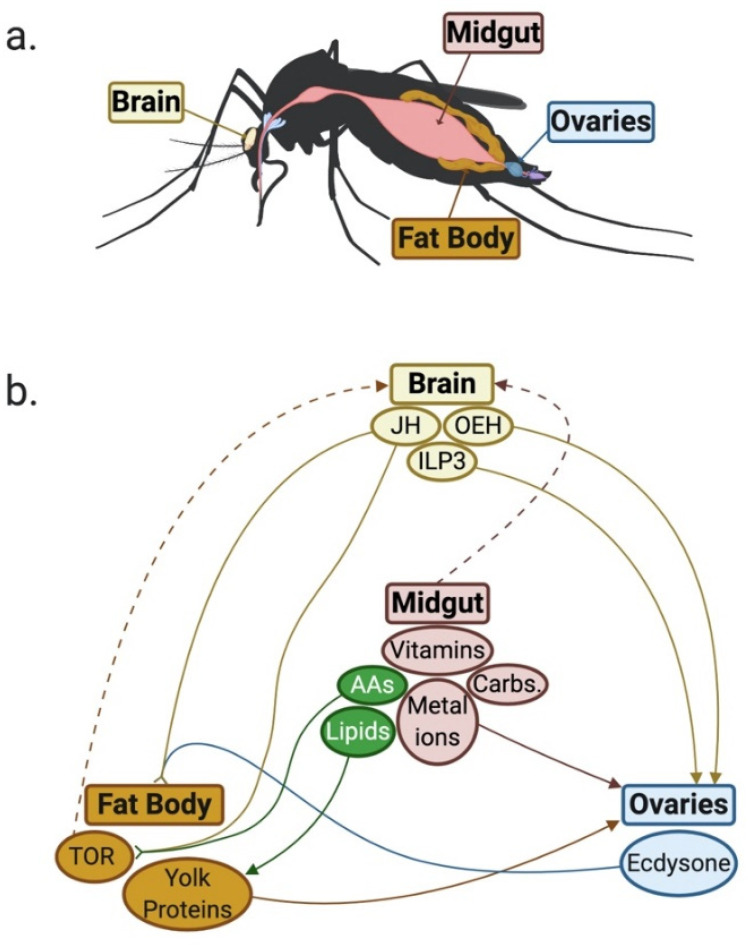
(**a**) Female *Aedes aegypti* organs involved in blood-meal digestion and subsequent egg production. (**b**) Female *Aedes aegypti* nutritional and hormonal signals stimulated by blood-meal. JH: juvenile hormone, OEH: ovary ecdysteroidogenic hormone, ILP3: insulin-like peptide 3, Trypsin prot.: Trypsin proteases, TOR: target of rapamycin, S6K: S6 kinase, Vg: vitellogenin, AAs: amino acids, Carbs.: carbohydrates. Green icons indicate metabolites that are modulated by the presence of *Wolbachia*. Solid arrows indicate metabolite release and delivery, solid arrows with splayed arrowheads indicates two metabolites working synergistically together and dotted arrows indicate upregulation/release of target metabolite. Figure created with BioRender.com.

In anautogenous female mosquitoes, ingestion of a vertebrate blood-meal activates multiple pathways that break down and deliver nutrients to the brain and reproductive organs (the fat body and ovaries; Figure 1a) required for production of eggs [63,73]. Egg production involves oogenesis (oocyte development in the ovaries) and vitellogenesis (production, secretion and deposition of yolk proteins from the fat body into developing oocytes), followed by ovulation, fertilisation, ovipositing, embryogenesis and chitinisation of eggshell. Oogenesis begins in the aquatic stages of development before arresting in adulthood [74,75], until ingestion of a blood-meal which activates mechanoreceptors and nutrient-sensitive receptors that release hormones; juvenile hormone (JH), the steroid hormone ecdysone and insulin-like peptides (ILPs) from the brain (Figure 1b) [76,77]. JH, ILP3 and amino acids work in the fat body to activate vitellogenesis via the nutrient-sensitive target of rapamycin pathway [63,78]. Ecdysone and ILP3 act in the ovaries to reactivate oogenesis and communicate with the fat body to ensure that egg yolk proteins are deposited into developing oocytes [74,75,79]. It is evident that blood-sourced nutrients are essential pathway stimulators that drive the release of hormones and activation of vitellogenesis and oogenesis. The nutritional status of *Ae. aegypti,* beyond what is received via the blood-meal, is also important since eggs cannot exogenously source nutrients, and all nutritional requirements for embryogenesis, as well as longevity, must be packaged during oogenesis [80]. After ovipositing, eggs undergo embryogenesis prior to entering a quiescent state. Embryogenesis involves the replication, growth and differentiation of cells to form a first instar larva encased within the eggshell [81,82].

As previously described, the presence of *Wolbachia* has been found to delay embryogenesis and decrease desiccation resistance and egg longevity. If the basis for decreased longevity of *Wolbachia*-infected eggs is altered nutritional availability, then a more informed and tailored diet could perhaps overcome this fitness effect.

### 5.2. Nutritional Requirements for Aedes aegypti and Drosophila Egg Production

#### 5.2.1. Amino Acids

The composition of blood-meal amino acids is critical for the activation of vitellogenesis and oogenesis pathways [77]. Amino acids required for vitellogenesis were identified by analysing the change in amino acid composition in mosquito hemolymph (tested in *Culex pipiens*) post blood-meal [83]. A mixture of 17 amino acids was then injected into adult female mosquito hemocoel and found to successfully stimulate oogenesis and mature egg development [84]. Attardo et al. [77] extended this work by removing individual amino acids from culture media and analysing how this impacts ecdysone activation of the vitellogenin gene. It was found that removing just one essential amino acid from the culture media reduces vitellogenin expression by more than 90%. This study identified essential, semi- and non-essential amino acids for successful vitellogenesis, based on the extent to which vitellogenin expression was affected when individual amino acids were removed (Table 2). In support of the role of competition for amino acids in *Wolbachia*-infected mosquitoes, Caragata et al. [69] found that the negative impact of *w*MelPop on *Ae. aegypti* fecundity and egg viability can be partially negated by supplementing the essential amino acids into sucrose. This suggests that increasing amino acid availability to *w*MelPop-infected mosquitoes can curb the pathogenic impact of this *Wolbachia* strain.

Studies conducted in *D. melanogaster* (which carry a natural *Wolbachia* infection) also demonstrate the importance of amino acids in insect egg production. Piper et al. [85] compared feeding *D. melanogaster* a holidic (chemically defined) diet and individually removed sterols, sugars, essential amino acids (that must be acquired from the diet), arginine only, isoleucine only, vitamins or metal ions. The removal of any of these components had a negative impact on fecundity, with the most significant decline observed when essential amino acids were removed, suggesting that amino acids are more likely to be sourced from the diet, whereas other components may be available from body stores. Another study in *D. melanogaster* found that under protein-deficient circumstances, the cell proliferation rate decreased in the ovaries while cell death increased, specifically during mitosis in the germanium and at the onset of vitellogenesis [86]. Furthermore, suppression of amino acid transporters in the fat body caused a reduction in egg laying and germline stem cells [78]. Therefore, if *Wolbachia* is utilising host amino acids, this could inhibit the signalling pathways and nutrients required for egg production, ultimately impacting on egg quality.

#### 5.2.2. Lipids

In addition to amino acids, lipids play an important role across insect survival and reproduction as they provide structure, energy and act as hormonal precursors [87,88]. Mosquitoes cannot perform de novo synthesis of sterols, a class of lipids; hence, they must be sourced from the larval diet and blood-meal [89,90]. Cholesterol is the main sterol required by mosquitoes, alongside 7-dehydrocholesterol, sitosterol and stigmasterol [90,91,92]. The role of cholesterol is to provide membrane stability and cellular signalling and act as a precursor of ecdysteroid hormone [87,93]. Studies in *Ae. aegypti* have shown that low teneral reserves of cholesterol negatively impact mosquito fecundity, which can be improved by supplementing the blood-meal with cholesterol micelles or low-density lipoproteins [94]. *Ae. aegypti* regulate the amount of lipids within individual mature oocytes to remain consistent across gonotrophic cycles, suggesting that if lipid availability is low, the number of eggs produced (i.e., fecundity) is likely to be affected [95]. More detailed analysis of oocyte lipid deposition has been conducted in *Drosophila* to show that when fed a low triglyceride diet, the percentage of ovarioles able to mature to the lipid deposition stage reduces; meanwhile, triglyceride levels within fully developed oocytes remain unaffected [96]. Additionally, Piper et al. [85] demonstrated that the removal of sterols from the *Drosophila* diet results in a minimal decrease in egg laying but a dramatic shortening of adult lifespan. While evidence suggests that a reduction in lipid availability is more likely to cause disruptions to mosquito survival and fecundity, further studies are required to determine how egg viability and longevity in *Ae. aegypti* is affected by a low lipid diet and whether *Wolbachia’s* utilisation of cholesterol, as demonstrated by Caragata et al. [69] and Geoghegan et al. [70], impacts these fitness factors.

It is important to consider that nutritional parasitism may also contribute to the antiviral state that *Wolbachia* induces in *Ae. aegypti* [97,98]. Studies have shown that viruses such as DENV are reliant on fatty acids produced by its host; therefore, competition for resources could also be occurring between virus, *Wolbachia* and mosquito host [99,100]. Although it is unlikely that feeding laboratory mosquitoes an altered blood-diet would affect the metabolic state of its offspring by the time they reach adulthood and are exposed to viruses, it would be prudent to assess this possibility by assaying the vector competence of mosquitoes and their offspring following diet supplementation. Such studies may also provide insight into the mechanisms that underlie the antiviral activity of *Wolbachia*.

#### 5.2.3. Other Nutrients

In addition to amino acids and lipids, other nutrient components, such as iron and sucrose, can increase the blood-feeding success and fecundity of *Ae. aegypti*. In *Wolbachia*-free mosquitoes, the presence of haemoglobin in an artificial blood formula increases fecundity and fertility [101,102]. In *Ae. aegypti*, while the majority of ingested blood-meal iron is excreted, the proportion of iron that is absorbed is mostly loaded onto ferritin and translocated to the ovaries and eggs [103]. Studies investigating the ability of red blood cells alone to induce successful vitellogenesis and produce viable eggs have been contradictory. Gonzales et al. [102] demonstrated that in *Wolbachia*-free *Ae. aegypti*, a blood-meal containing only haemoglobin or red blood cells resulted in no ovarian development or egg laying, while a diet based on bovine serum albumin (the protein component of blood) rescued this. Meanwhile, Dutra et al. [104] separated blood components and fed *Wolbachia*-infected *Ae. aegypti* whole blood, plasma only and red blood cells only and found that while red blood cells did result in a decrease in fecundity, egg production was successful and egg viability was better than plasma only derived eggs. This difference could be due to a *Wolbachia*–iron interaction, as *Wolbachia* has been shown to assist with iron homeostasis and reduce oxidative stress in *Drosophila* [105,106]. Regarding sucrose, which is an essential form of carbohydrate used for energy production [73,107], adequate levels of energy reserve provided by sugar feeding are also very important for optimal egg production. If energy stores are depleted, a blood-meal is not sufficient to initiate egg development [107]. *Wolbachia* is not likely to be impacting iron or sucrose availability to its host as only the most virulent *Wolbachia* strain, *w*MelPop-CLA, is consistently linked to disruption in fecundity [32,49] and adult longevity [13,50] (Table 1), and *Wolbachia* infection has not been shown to negatively impact attraction to a blood-meal [51]. Overall, while it is important to understand the role of all nutrients, it is evident that deficiencies of protein and lipids are the most significant causes of disruptions in egg production.

## 6. Conclusions and Recommendations

It has been well documented that various *Wolbachia* strains reduce *Ae. aegypti* egg survival during quiescence. Here, we have explored the potential reasons behind this effect and considered how nutrient supplementation improves *Wolbachia*-infected egg viability. Egg production in *Ae. aegypti* is stimulated by the ingestion of vertebrate blood and is significantly impacted by the nutritional composition of the blood-meal as well as its host. If *Wolbachia* is acting as a nutritional parasite and impacting essential nutrient availability within mosquitoes, egg development could be impeded. This implies that the blood-meal of *Wolbachia*-infected *Ae. aegypti* requires adequate levels and proportions of essential nutrients for optimal egg development. Further work is required to understand the different changes in nutritional availability between *Wolbachia-*infected and uninfected eggs, particularly over extended quiescent periods, and to investigate potential changes to mass insect production methods, such as diet supplementation, that could alleviate *Wolbachia*-specific fitness effects. By doing so, mass production of *Wolbachia-*infected mosquitoes could be more productive, yielding higher quality eggs and facilitating the expansion of *Wolbachia*-*Ae. aegypti* release programs to reduce vector-borne diseases globally.

## Figures and Tables

**Table 1:** Fitness impacts of *w*Mel, *w*AlbB and *w*MelPop-CLA on *Aedes aegypti* fitness, relative to *Wolbachia*-free *Aedes aegypti.*

*Wolbachia* Strain	Fecundity	Larval Development	Adult Female Longevity	Blood Feeding Success	Egg Longevity
*w*Mel	Minor or no negative impact [32,33,40,47]	No negative impact [32]	No negative impact [15,33]	No negative impact [51]	Decreased [15,47,51] or no negative impact [33]
*w*AlbB	No negative impact [33,46,47]	No negative impact [46]	Minor or no negative impact [33,46,47]	No negative impact [51]	Decreased [33,46,47] or no negative impact [33]
*w*MelPop-CLA	Decreased (age associated) [32,49,50]	Survival not impacted. Delay in male pupation and female 4th instar larval development [49]	Decreased [13,50]	Decreased [48], exacerbated with age [49]	Decreased [32,49,50]

**Table 2:** Essential, semi-essential and non-essential amino acids required for successful egg production. Data from Attardo et al. [77].

Essential	Semi-Essential	Non-Essential
Leucine	Cysteine	Tyrosine
Tryptophan	Glycine	Aspartic acid
Methionine	Isoleucine	Serine
Valine		Proline
Histidine		Glutamine
Lysine		Alanine
Phenylalanine		Glutamic acid
Arginine		
Asparagine		
Threonine		
